# Primary acrocyanosis

**DOI:** 10.1002/jgf2.416

**Published:** 2021-02-02

**Authors:** Yuki Takeuchi, Junji Tsukagoshi

**Affiliations:** ^1^ Teine Family Medicine Clinic Sapporo Japan; ^2^ Teine Keijinkai Hospital Sapporo Japan

**Keywords:** family medicine, acrocyanosis

## Abstract

Primary acrocyanosis is a benign condition characterized by persistent blue discoloration of the peripheral extremities caused by vasospasm.

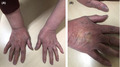

A previously healthy 71‐year‐old Japanese woman presented to the clinic with a 2‐day history of persistent blue discoloration of the hands. Her symptoms presented while shoveling snow with bare hands in Hokkaido in January. Although warmth slightly alleviated the discoloration, the change in the skin color persisted. She denied any symptoms of pain, paralysis, paresthesia, morning stiffness, arthralgia, weight change, or red and white skin color changes. The patient did not have a history of smoking or family history of cancer or rheumatic disease. On physical examination, she was afebrile with pulse rate of 88 beats per minute, blood pressure of 138/96 mm Hg, and oxygen saturation of 98% on room air. Clinical examination revealed symmetric blue discoloration of the hands. The blue discoloration transiently disappeared on application of finger pressure (Figure 1). The auscultatory and neurological findings and bilateral Allen test were unremarkable. No edema, ulcers, or other dermatological changes were noted. The results of laboratory tests revealed normal complete blood count, liver and kidney function, electrolytes, blood sugar, and coagulation system. The screening tests for antinuclear antibodies, rheumatoid factors, anticardiolipin antibodies, antiphospholipid antibodies, and lupus anticoagulants were negative. All cancer screening tests recommended by the National Cancer Center Japan were normal.^1^ We made a diagnosis of acrocyanosis by ruling out chilblain, vaso‐occlusive disorders, and Raynaud's phenomenon from the patient's history and skin findings. Moreover, the history, physical examination, and laboratory tests did not reveal the secondary causes of acrocyanosis (connective tissue diseases, neoplasms, spinal cord injury, eating disorders, or drugs). Therefore, we established a diagnosis of primary acrocyanosis.

**FIGURE 1:**
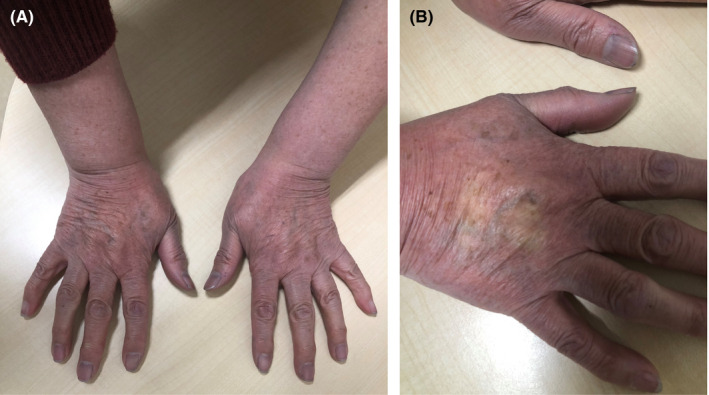
A, Blue discoloration of the hands. B, Disappearance of the blue discoloration transiently on application of finger pressure

Acrocyanosis is a condition characterized by persistent blue discoloration of the peripheral extremities caused by vasospasm.^2^, ^3^, ^4^ Exposure to cold temperature is the most common trigger. The skin changes frequently persist during winter and even in summer.^4^ Acrocyanosis is much less common than other acrosyndromes (Raynaud's phenomenon, chilblain, acrorygosis, blue finger syndrome, and erythromelalgia); however, the actual prevalence is unknown.^4^ Acrocyanosis should be differentiated from Raynaud's phenomenon, chilblain, and vaso‐occlusive disorders. While Raynaud's phenomenon is characterized by a paroxysmal episode of triphasic or biphasic color change of the fingers and toes, acrocyanosis shows persistent blue discoloration. Chilblain is also developed after cold exposure of the digits with erythematous and purplish discoloration; however, other symptoms, such as itching, burning, and pain, are often presented in contrast to acrocyanosis. Acrocyanosis shows normal capillary oximetry, unlike vaso‐occlusive disorders. This finding helps to eliminate vaso‐occlusive disorders.^4^ Acrocyanosis is categorized into primary and secondary acrocyanosis. Secondary acrocyanosis has various underlying conditions, such as connective tissue diseases, neoplasms, spinal cord injury, eating disorders, or drugs.^3^, ^4^, ^5^ Secondary acrocyanosis is often asymmetrical and is associated with pain and tissue damage compared to primary acrocyanosis.^3^ This patient had typical skin features of primary acrocyanosis, and she did not have any history or laboratory findings indicating secondary causes. Hence, we established the diagnosis of primary acrocyanosis. There is no standard curative treatment for primary acrocyanosis. Reassurances, gloves, avoidance of exposure to cold, smoking cessation, alpha‐blockers, and calcium channel blockers are recommended.^4^ In this case, the symptoms of the patient persisted for approximately two months despite lifestyle modification. Thus, we prescribed her with nifedipine 10 mg. Several days after the initiation of medication, the skin discoloration was resolved (Figure 2). Seasonal change to spring may also have contributed to the improvement of the symptoms.

**FIGURE 2:**
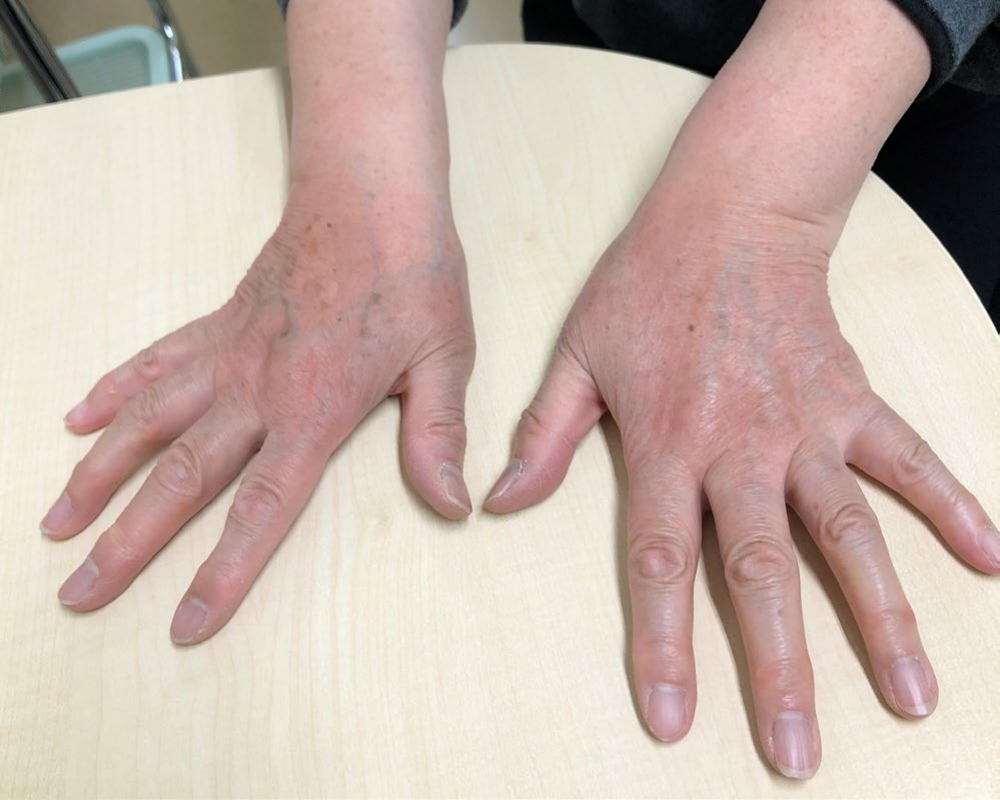
Disappearance of the blue discoloration two months after the onset of symptoms

Inspection and history‐taking are important factors in the diagnosis of acrocyanosis. Thus, primary care physicians should be aware of the clinical features of acrocyanosis. More importantly, primary acrocyanosis is a diagnosis of exclusion. Careful differentiation of primary acrocyanosis from secondary acrocyanosis is necessary because acrocyanosis can be an important sign indicating an underlying serious condition.

## CONFLICT OF INTEREST

The authors declare no conflict of interest for this report.

## INFORMED CONSENT

Informed written consent was obtained from the patient for publication of this report and any accompanying images.
